# Automated surveillance for surgical site infections (SSI) in hospitals and surveillance networks–expert perspectives for implementation

**DOI:** 10.1186/s13756-024-01505-2

**Published:** 2024-12-23

**Authors:** Luisa A. Denkel, Isabelle Arnaud, Manon Brekelmans, Mireia Puig-Asensio, Hoger Amin, Sophie Gubbels, Pernille Iversen, Mohamed Abbas, Elisabeth Presterl, Pascal Astagneau, Stephanie van Rooden, PRAISE SSI working group, Isabelle Arnaud, Manon Brekelmans, Mireia Puig-Asensio, Hoger Amin, Sophie Gubbels, Mohamed Abbas, Elisabeth Presterl, Pascal Astagneau, Stephanie van Rooden, Seven Aghdassi, Heike Düsseldorf, Karl Mertens, Maaike S. M. van Mourik, Suzanne D. van der Werff

**Affiliations:** 1 Institute of Hygiene and Environmental Medicine, Charité Universitätsmedizin Berlin, Humboldt-Universität Zu Berlin and Berlin Institute of Health, Hindenburgdamm 27, 12203 Berlin, Germany; 2 National Reference Center for the Surveillance of Nosocomial Infections, Charité Universitätsmedizin Berlin, Humboldt-Universität Zu Berlin and Berlin Institute of Health, Berlin, Germany; 3 Centre for Prevention of Healthcare-Associated Infections, Assistance Publique-Hôpitaux de Paris, Paris, France; 4 Centre for Infectious Diseases Control, National Institute for Public Health and the Environment, Bilthoven, The Netherlands; 5 Department of Medical Microbiology and Infection Control, University Medical Centre Utrecht, Utrecht, The Netherlands; 6 Department of Infectious Diseases, Bellvitge University Hospital, L’Hospitalet de Llobregat, Barcelona, Spain; 7 Centro de Investigación Biomédica en Red de Enfermedades Infecciosas (CIBERINFEC¸ CB21/13/00009), Instituto de Salud Carlos III, Madrid, Spain; 8 Institut d’Investigació Biomèdica de Bellvitge (IDIBELL), L’Hospitalet de Llobregat, Spain; 9 Department of Data Integration and Analysis, Staten Serum Institut, Copenhagen, Denmark; 10 Regionernes Kliniske Kvalitetsudviklingsprogram, Aarhus, Denmark; 11 Infection Control Programme and WHO Collaborating Centre on Infection Prevention and Control and Antimicrobial Resistance, Geneva University Hospitals, Geneva, Switzerland; 12 MRC Centre for Global Infectious Disease Analysis, Jameel Institute, School of Public Health, Imperial College London, London, UK; 13 Department of Hospital Epidemiology and Infection Control, Medical University of Vienna, Vienna, Austria; 14 Institute of Epidemiology and Public Health, INSERM, Sorbonne University, Paris, France; 15 Berlin Institute of Health at Charité-Universitätsmedizin Berlin, BIH Biomedical Innovation Academy, BIH Charité Digital Clinician Scientist Program, Berlin, Germany; 16 Service of Healthcare-Associated Infections and Antimicrobial Resistance, Sciensano, Brussels, Belgium; 17 Department of Medicine Solna, Division of Infectious Diseases, Karolinska Institutet, Stockholm, Sweden; 18 Department of Infectious Diseases, Karolinska University Hospital, Stockholm, Sweden

**Keywords:** Surgical site infection, Automated surveillance, Barriers, Experiences, Expert recommendations

## Abstract

**Background:**

This work aims at providing practical recommendations for implementing automated surveillance (AS) of surgical site infections (SSI) in hospitals and surveillance networks. It also provides an overview of the steps, choices, and obstacles that need to be taken into consideration when implementing such surveillance. Hands-on experience with existing automated surveillance systems of SSI (AS SSI systems) in Denmark, France, the Netherlands and Spain is described regarding trend monitoring, benchmarking, quality control, and research for surveillance purposes.

**Methods:**

Between April and October 2023, specific aspects/options of various surveillance purposes for AS SSI were identified during regular meetings of the SSI working group in the PRAISE (Providing a Roadmap for Automated Infection Surveillance in Europe) network. Expert discussions provided the basis for this perspective article.

**Results:**

Decisions for implementation of AS SSI systems highly depend on the purpose of the surveillance. AS SSI systems presented here differ according to study population, setting, central or local implementation; the level of automation, design, and the data sources used. However, similarities were found for the rationales of automation, design principles and obstacles that were identified. There was consensus among all the experts that shortcomings in interoperability of databases, limited time, a want of commitment on the part of stakeholders, and a shortage of resources for information technology (IT) specialists represent the main obstacles for implementing AS SSI. To overcome obstacles, various solutions were reported, including training in the development of AS systems and the interpretation of AS SSI results, early consultation of end-users, and regular exchanges between management levels, IT departments, infection prevention and control (IPC) teams, and clinicians.

**Conclusion:**

Clarity on the intended application (e.g. purpose of surveillance) and information on the availability of electronic and structured data are crucial first steps necessary for guiding decisions on the design of AS systems. Adequate resources for IT specialists and regular communication between management, IT departments, IPC teams, and clinicians were identified as essential for successful implementation. This perspective article may be helpful for a wider implementation of more homogeneous AS SSI systems in Europe.

**Supplementary Information:**

The online version contains supplementary material available at 10.1186/s13756-024-01505-2.

## Introduction

Surgical site infections (SSIs) are among the most prevalent healthcare-associated infections (HAI) [1]. According to the World Health Organization (WHO), SSI are caused by microorganisms that enter the body, usually through incisions during surgery, but they may also be caused by endogenous microorganisms [2]. Surveillance is known to effectively reduce SSI rates [3, 4]. As such, surveillance is one of the key components in effective infection prevention and control (IPC) programs [5]. Automated surveillance of SSI (AS SSI) can be used to achieve various objectives, so-called “surveillance purposes,” that are pre-determined by those implementing AS SSI—individual hospitals, hospital networks, and national or international public health institutes. Ideally, surveillance should be performed on all surgical procedures performed in a hospital [6]. However, surveillance activities are still mainly manual, with data collection necessitating a considerable outlay of labour time and man power, especially for surgical staff and IPC teams. At the same time, in Europe as well as other parts of the world, electronic routine care data has become increasingly available from hospital information systems (HIS), such as electronic health records (EHR), laboratory information management systems (LIMS), pharmacy antibiotics supplier systems, and hospital discharge databases (HDD). This could serve as the optimal basis for automated surveillance (AS). Using this approach, manual assessments are replaced by automated ones based on routine data available in HIS. This assumes the collection of denominator data, characteristics, and risk factors; the selection of procedures under surveillance; and the detection of HAI. Full AS comprises all steps of surveillance, including data collection and the determination of HAI status without human intervention or interpretation, while semi-AS combines automation with chart review of selected cases and manual confirmation when detecting and defining a HAI [7].

Development and implementation of AS is challenging. Many aspects have to be addressed in order to achieve a robust, sustainable, and transparent approach. These include determination of appropriate definitions, the availability of data, algorithm development and validation, governance, and the IT environment, as well as data security and protection [8, 9]. The importance of clinical relevance, buy-in and actionable data, large scale standardization, reliability over time, and timeliness of AS depend on the surveillance purpose, as previously illustrated [7]. Although it has been envisioned for decades, practical implementation of AS has occurred mainly in research settings or single centres [10, 11]. Large scale implementation has been achieved only recently [12–16].

To facilitate implementation of large-scale automated surveillance, van Mourik et al. developed a roadmap called “Providing a Roadmap for Automated Infection Surveillance in Europe” (PRAISE) [8]. This roadmap developed definitions, terminology, and key issues concerning design, targets, and approaches to implementing AS [8].

In 2019, Verberk and colleagues conducted a survey to map the current state of AS systems in Europe and provided detailed information on three existing European AS systems [11]. They reported that 10 operational AS systems for SSI were in current use. Five of them had been implemented on the hospital level and another five on the surveillance network level. Verberk identified items related to methodology, algorithms, data sources, and targeted HAIs that should be reported in publications on automated surveillance systems in order to facilitate a more widespread development of AS along with comparability between surveillance systems [11].

Automated surveillance for SSI has been shown by numerous publications to be feasible, reliable, highly sensitive, and successful in reducing workload [15–28]. However, scientific literature with detailed descriptions of existing AS systems that specifically focuses on SSI is scarce. This perspective article aims to fill this gap by describing the key aspects for implementation of AS SSI in hospitals and surveillance networks based on expert discussions and experience. As a starting point, we elaborated basic requirements of AS SSI systems for various surveillance purposes. Subsequently, we present examples of different systems, one per surveillance purpose, with detailed information on the design and implementation of AS SSI in each.

### How this article should be read

This work focusses on four different surveillance purposes and provides an overview of AS SSI systems that have been successfully implemented in Europe. This information can be used by readers who plan to implement AS SSI systems in their hospital or surveillance network, as advice on specific requirements and to help identify transferability and whether the systems can be adapted to meet individual needs. Those interested in specific surveillance purposes and examples of AS SSI systems presented here will find further information in the Supplementary material.

## Methods

### Expert group discussions and process of data acquisition

“Providing a Roadmap for Automated Infection Surveillance in Europe” (PRAISE) is both the name and the goal of a network funded by the Joint Programming Initiative on Antimicrobial Resistance (JPIAMR) Network Call on Surveillance from 2019 to 2020 [8]. Today, the PRAISE network continues its activities as an unfunded collaboration hosting various working groups that combine activities and experience to develop automated HAI surveillance (e.g. formulation of suitable definitions and algorithms, standardization of data sources). The PRAISE SSI working group currently consists of more than 15 members from hospitals, universities, and public health institutes in Western Europe, including Austria, Belgium, Denmark, France, Germany, the Netherlands, Sweden, and Switzerland. The SSI working group’s purpose is to coordinate AS SSI development in Europe by defining and recommending solutions concerning methodology, definitions and algorithms, describing the potential impact of alternative choices and possible collaborative development of algorithms.

This work is based on discussions in the PRAISE SSI working group that were held between April and October 2023, in which specific aspects and options for various AS SSI surveillance purposes were identified. Consensus among working group members regarding the basic requirements for automated SSI surveillance was reached in discussion until an agreement was achieved. Subsequently, a project group was formed whose task was to generate a document to assist future implementation of AS SSI in hospitals and surveillance networks. Existing AS SSI systems were identified for each surveillance purpose. All members of the project group reported their own practical experience with their AS SSI system. User surveys (SPICMI), evaluation studies on implementation (PREZIES–PAS ORTHO), and studies on additional scientific research outcomes (SPICMI) were also used, when available. Detailed descriptions of the AS SSI systems were provided by members of the project group and their colleagues who had been actively involved in the development, maintenance, management, or use of such systems and who allowed their input to be made public.

## Results

### Consensus for basic requirements for automated AS SSI

The PRAISE SSI working group elected to focus on the four surveillance purposes for AS SSI that are most relevant for the European setting: (1) trend monitoring, (2) benchmarking, (3) internal quality control/improvement, and (4) research. An overview of all surveillance purposes and their basic requirements for automation can be found in Table 1. As outlined there, the surveillance purpose has a direct impact on the selection of the procedures, algorithms, and risk factors considered. It is important that the latter be collected and are, thus, available and comparable with source data in all participating centres (if this concerns more than one healthcare facility (HCF)). At the same time, AS SSI that is performed in hospitals solely for internal infection control and quality improvement or for individual research purposes allows greater flexibility. For research, choices are guided primarily by the research questions and data available for research purposes. However, if comparability over time is important and comparability to a (national) reference standard is desirable, there will be constraints on the specifications of automated surveillance and source data [27].

**Table 1:** Surveillance purposes for AS SSI and basic requirements for automation

Aim of surveillance/use-case	Target audience/beneficiaries	Data qualifications	Surveillance population	Semi/full AS	Case definitions	Algorithms for case ascertainment	Specifications source data	Risk factors	Feedback information (to target audience)
Trend monitoring	Reference centers; policy advisors, hospital IPC teams, HCWs, management; patients	> 1 hospital, consistent over time and between hospitals	Automated selection feasible in all hospitals; widely applied procedures; international comparability	Both	Actionable; comparable between hospitals (and internationally); source data widely available	Source data available at all hospitals; algorithm applicable and accepted; performance explainable	Preferably structured and standardized; not too sensitive for changes over time (too specific)	Sufficient source data electronically available for adjustment	Clear, explainable, anonymous
Benchmarking	Reference centers; policy advisors, hospital IPC teams, HCWs, management; patients	> 1 hospital, consistent over time and between hospitals	Automated selection feasible within all hospitals; widely applied procedures	Both	Actionable; comparable between hospitals; source data widely available	Source data available at all hospitals; algorithm applicable and accepted; performance explainable	Preferably structured and standardized; not too sensitive for changes over time (too specific)	Sufficient source data electronically available for adjustment	Clear, explainable, actionable for HCW, anonymous
Internal quality control and improvement (/plan-do-check-act)	Hospital IPC teams, HCWs, departments of quality improvement, patients	1 hospital, consistent over time; comparison with reference data^a^	Automated selection feasible	Both	Actionable; source data available; comparable between hospitals^1^	Source data available; validated algorithm with good performance, explainable	Preferably structured; not too sensitive for changes over time (too specific)	Sufficient source data electronically available for adjustment, possible, if needed (not essential)	Timely, clear, explainable, actionable for HCW
Research (into risk factors, interventions and epidemiology of HAI)	Scientific community; Hospital IPC teams, HCWs, management; departments of quality improvement; patients; reference centers; policy advisors	Depends on research question; ≥ 1 hospital(s)	Available for research purposes (informed consent or opt out procedures)	Both	Relevant for aim of study; information available and accessible for research purposes	Source data available for research purposes; knowledge algorithm performance	Flexible	Which information is needed and should be electronically available depends on research question	Valid, generalizable

AS; automated surveillance, IPC; infection prevention and control, HCW; Healthcare workers

^a^specifically, relevant for quality control with comparison to reference data over plan-do-check-actTrend monitoring—application of the Danish healthcare-associated infections database (HAIBA)

How the requirements of Table 1 can support decision makers who plan to implement AS SSI in their hospital or surveillance network, will be further illustrated by one practical example for each surveillance purpose presented here (Table 2).

**Table 2:** AS SSI systems presented by this work

AS SSI system	Danish Healthcare-associated infections database (HAIBA)	Dutch AS SSI system for hip and knee replacement (PREZIES–PAS ORTHO)	Spanish local hospital AS SSI system (No name determined)	French surveillance and Prevention of Infection in Surgery and Interventional Medicine (SPICMI)
Institution hosting the AS SSI system, country	Danish National Institute for Public Health, Statens Serum Institut, Denmark	Dutch National Institute for Public Health and the Environment (RIVM), the Netherlands	Bellvitge Hospital, Spain	Centre for Prevention of Healthcare-Associated Infections (CPias) and Santé Publique France (SpF), Paris, France
Aim of surveillance/surveillance purpose	National trend monitoring, internal quality control, research	National trend monitoring, risk factor monitoring, benchmarking	Hospital trend monitoring, internal quality control and improvement	SSI identification for targeted specialties, benchmarking, internal quality control, research
Aim of automation	Offer national, continuous surveillance of HAIs and provide a standardized data basis for infection control efforts and research	Reduction of workload and improvement of data quality by means of increased standardization of surveillance	Reduce the workload of surveillance	Reduce workload of surveillance
Stakeholders involved	Department of Data Integration and Analysis at Statens Serum Institut, clinical societies, RKKP, surgeons, hospital management, IPC teams, researchers	Medical microbiologists, IPC specialists, orthopedic surgeons, IT and legal specialists, scientific associations, umbrella organizations for hospitals	Frontline stakeholders (IPC teams, surgeons, nurses), hospital management, IT teams, pharmacists, microbiologists, healthcare coding department	AS SSI teams at CPias, IPC teams, surgeons, IT teams, nurses, hospital management, healthcare coding department
Setting, e.g. hospital, hospital network	National level, all hospitals in Denmark	Voluntarily participating hospitals	A single hospital	All voluntary public and private French hospitals practicing surgery, each participating HCF selects at least one surgical specialty and at least one associated procedure for inclusion during the first 6 months every year
Level of automation	Fully automated	Semi-automated	Semi-automated	Semi-automated
Implementation approach	Centrally implemented	Locally implemented	Locally implemented	Locally implemented
Target / Surveillance population with inclusion criteria	All Danish residents undergoing primary hip or knee arthroplasty procedures	Patients in participating hospitals undergoing primary hip or knee arthroplasty procedures	Adult patients undergoing cardiac surgery and prosthetic knee and hip replacements	Patients undergoing digestive surgery, gyneco-obstetrics surgery, neurosurgery, coronary surgery, orthopedic surgery and/or urologic surgery
Selection of patient population under surveillance	Fully automated, using procedure codes based on the Nordic Medico-Statistical Committee (NOMESCO) Classification of Surgical Procedures [31]	Fully automated, patient selection based on procedure codes in EHR data	Currently partially automated, population under surveillance (denominator data) selected by specific ICD-10 codes, completeness and reliability currently manually verified	Partially automated, HDD for procedures and diagnosis (ICD-10 codification), some variables extracted from the HDD required post-hoc recoding or manual editing
SSI types/definition targeted	Deep/incisional SSI	Deep/incisional SSI	Deep and organ/space SSI	Superficial, deep and organ/space SSI
Follow-up period	2–90 days after the index operation and up to 365 days	90 days, while the algorithm performs monitoring until 120 days post-surgery	90 days, while the algorithm performs monitoring until 120 days post-surgery	30 days according to usual definition, extended to 90 days for prosthetic surgery (orthopaedic, cardiac and breast)
Data sources for patient selection and algorithms	MiBa, NPR (diagnosis and procedure codes, operation dates), SOR (geographical data)	EHR (Demographic/administrative data, procedures, antibiotic prescriptions) and Laboratory information system (LIMS; culture orders and results)	Hospital data warehouse (microbiological data; radiological data; pharmacy data) and the minimum basic set of discharge data (CMBD, National Health System) with sociodemographic and administrative data;	HDD with demographic/administrative data, comorbidities, etc.), microbiological data base, local IT systems (i.a. NNIS risk scores, Altmeier wound classification)
Phase of the automated surveillance system	In current use	In current use/ upscaling phase	Development phase / in current use	In current use
Quality management / maintenance	Quarterly meetings between surgeons, hospital management, IPC teams	Manual, user group meetings, individual guidance and training, evaluation of the strategy in 5 frontrunner hospitals for strategy improvement	Regular audits of the process, annual monitoring of accuracy of data extraction and algorithm performance, comprehensive version control and documentation summarizing design decisions, algorithms and methodologies	Electronic platform for hospital data recording, quarterly steering committee meetings, all guidelines available on SPICMI website, monthly webinars, research projects for improvement of surveillance performance (e.g. choice of risk factors, algorithm effectiveness)
Year of implementation	2015	2022	2024	2021

AS, automated surveillance. CMBD, Minimum Basic Data Set (with the Spanish acronym). CPias, Centre for prevention of healthcare-associated infections. ia, inter alia. ID, infectious disease. IPC, infection prevention and control. IT, information technology. HAI, healthcare-associated infection. HAIBA, Danish Healthcare-associated infections database. HCF, healthcare facility. HCW, healthcare worker. EHR, electronic health records. HDD, Hospital discharge databases HIS, hospital information system. LIMS, laboratory information system. MiBa, Microbiology Database. NNIS, National Nosocomial Infection Surveillance System. NPR, National Patient Register. PAS ORTHO, Dutch AS SSI system for hip and knee replacement. PREZIES, Preventie van zeikenhuisinfecties door surveillance. RIVM, Rijksinstituut voor Volksgezondheid en Milieu. RKKP, the Regional Clinical Quality Programme. SOR, System operation regions. SpF, Santé Publique France. SPICMI, Surveillance and Prevention of Infection in Surgery and Interventional Medicine. SSI, surgical site infection

## Trend monitoring – one application of the Danish healthcare-associated infections database (HAIBA)

### Purpose of surveillance and the surveillance population

An operational national AS SSI system was successfully implemented as part of HAIBA by the Danish public health institute, Statens Serum Institute, in 2016 [11, 29]. In HAIBA, AS SSI specifically monitors deep SSI following orthopaedic knee or hip surgery and is used for various purposes including trend monitoring, internal quality control, and research. Here, we will focus on the surveillance purpose “trend monitoring”.

The surveillance population consists of all residents of Denmark who have undergone a total hip and/or knee arthroplasty (Table 1). The aim of automation was to offer national, continuous surveillance of HAIs and to provide a standardized data basis for infection control efforts and research.

### Design principles, implementation approach, selection of algorithm, and level of automation (full/semi)

The key principles behind HAIBAs design are full automation, national coverage, and rule-based algorithms for the classification of SSI.

A key aspect of the implementation approach for HAIBA was the decision to develop the surveillance system internally at Statens Serum Institut, rather than outsourcing the programming to private providers. This represents an example of centrally implemented AS. With this approach, the team behind HAIBA can maintain and further develop systems tailored to their specific needs to increase sustainability, usefulness, and acceptance from users.

The following decisions, considerations, and compromises were made regarding fully automated surveillance on a national scale:

1. Full automation, using only information that is available electronically and that is accessible through a central database or register.
2. A simple algorithm to avoid large variability in source data between hospitals to enable the creation of a rule-based algorithm that can be applied to data from all hospitals in the country.
3. Ascertaining performance of the algorithm and completeness of data sources by the coordinating center.
4. Exclusion of superficial SSIs from the case-definition.
5. Non-inclusion of information on the pre- or post-operation use of medication, prophylaxis, or the use of antimicrobial prescriptions to treat the SSI unless national data can be linked to HAIBA.
6. HAIBA’s trend monitoring for SSIs may represent a conservative underestimate of the actual number of SSIs that occur due to the fact that identification of SSI cases is based on microbiological culture results; as such, SSI cases with negative cultures (due to prophylaxis) will not be detected.

With this fully automated approach, trend monitoring is more consistent and avoids inter-clinician and inter-case discrepancies arising from subjective evaluations. Trend monitoring of SSIs in HAIBA is made publicly available on an aggregated level [30].

A HAIBA steering committee was established for development and implementation. Members of the steering committee include representatives of the Ministry of Health and Elderly, the Danish Regions, the Danish Health and Medicines Authority, and various departments at Statens Serum Institut, which were responsible for project management. To ensure that stakeholders on the clinical level considered the system meaningful and acceptable, a stakeholder group was established and a workshop was held to provide specific advice on HAIBA.

### Requirements, data sources, and definitions

The basis for trend monitoring of SSI in HAIBA is the total microbiological test results submitted by departments of clinical microbiology to the Danish Microbiology Database (MiBa) together with procedure and diagnosis codes from the National Patient Register (NPR). In HAIBA, deep SSI following total knee and hip arthroplasty operations are identified using infection criteria applied to microbiological test results from MiBa, procedure and diagnosis codes (International statistical classification of diseases, 10th revision, ICD-10) from NPR and geographical information from system operation regions (SOR).

Primary knee and hip arthroplasty (THA/TKA) are defined using procedure codes based on the Nordic Medico-Statistical Committee (NOMESCO) Classification of Surgical Procedures [31]. Additional details can be found in Supplementary material.

### Implementation, maintenance and operationalization

In September 2011, implementation of HAIBA was commenced with a three-month pilot study in two hospitals from two different Danish regions. The aim of the pilot study was to determine whether HAIs can be monitored by linking existing data.

Maintenance is crucial when data formats or registration practices in the data sources can change on short notice without the knowledge of the third-party service provider, the Danish Public Health Institute. For this purpose, quarterly meetings are organized with an advisory group of representatives primarily IPC professionals from each region to discuss recent developments and surveillance purposes for the surveillance data and to receive feedback on end-user experience. HAIBA results are used by actors responsible for IPC at different national and regional levels to monitor trends in SSI incidence, estimate the disease burden, and identify areas requiring vigilance or action in the orthopaedic field. Therefore, the HAIBA team is in dialogue with various clinical societies, including the Danish Society for Orthopaedic Surgery, as well as the Regional Clinical Quality Programme (RKKP), which maintains the quality registries, including those for hip and knee arthroplasty [32]. Trend monitoring of SSI in HAIBA is publicly available on an aggregated level [30].

The Central Authority for Infection Control, also part of the Statens Serum Institut, formulates national infection control guidelines and works actively with hospitals to improve IPC. This Authority meets with the HAIBA team on a monthly basis to discuss the use of HAIBA and new developments.

### Obstacles and challenges

There are several challenges for AS SSI in HAIBA. First, maintenance of HAIBA requires continuous attention and can pose hurdles, particularly in keeping the system updated as clinical and registration practices change. For example, an increase of up to 30% in the relative proportion of uni-compartmental knee replacement resulted in a change in the procedures under surveillance and a specification of procedure codes. This highlights the need for close communication with stakeholders, such as the AS SSI team at Statens Serum Institut, and end-users, such as experts from the clinical quality programme mentioned above (RKKP), to ensure that the surveillance system adapts in a timely fashion to clinical reality.

Furthermore, HAIBA is very well equipped for trend monitoring, but validation of results on the local level is limited and, although clinicians have expressed a need for actionable data, aggregated results are not applicable to local quality improvement. In addition, lengthy legal procedures were required to access and utilize databases and registers that contained information relevant for HAI surveillance. This issue has recently been addressed by a change of national law. Since August 2023, the Statens Serum Institut is allowed to share data on an individual level with the Danish Regions. Consequently, this case data, in addition to aggregated-level data, offers IPC experts and orthopaedic surgeons greater insight into the courses of treatment of individual patients. HAIBA data can be linked with additional information from patient-specific medical records and clinical history using a unique patient identifier. The full potential of this data has yet to be explored in the Regions.

## Benchmarking: one application of the Dutch AS SSI system following orthopedic surgery (PREZIES PAS ORTHO)

### Purpose of surveillance and the surveillance population

In 2022, the PREZIES PAS ORTHO (**P**reventie van ziekenhuisinfecties door surveillance, **A**utomating **S**urveillance of SSI after **ortho**pedic surgery) project for national implementation of semi-automated surveillance of deep SSIs after hip and knee replacement was launched by the Dutch National Institute for Public Health and the Environment (Rijksinstituut voor Volksgezondheid en Milieu, RIVM) [14, 33].

PREZIES PAS ORTHO’s purpose is to monitor national trends, associated risk factors and to provide individual feedback and benchmark results to hospitals with the overall aim of reducing the number of infections. Here, we will focus on the surveillance purpose “benchmarking”. The goals of automation were defined as a reduction of workload and the improvement of data quality by means of increased standardization of surveillance.

SSI after hip and knee replacement was selected for automation due to the following reasons: (1) an algorithm had been developed and validated in Dutch hospitals; (2) the provision of care is reasonably standardized, thereby reducing the complexity of automating surveillance; and, (3) with a high number of procedures and low incidence of SSIs, these procedures had high likelihood of reducing workload. Taken altogether, they were considered “low hanging fruit”.

### Design principles, implementation approach, selection of algorithm and level of automation (full/semi)

PREZIES PAS ORTHO was the result of a preparatory process, guided by the PRAISE roadmap with accompanying guidelines on IT and governance aspects [8, 9, 34]. Throughout the development process, extensive stakeholder consultation was carried out with experts from the RIVM and hospitals, including medical microbiologists, IPC specialists, orthopaedic surgeons, IT and legal specialists, scientific associations, and umbrella organizations for hospitals.

A methodology that is feasible, acceptable, and endorsed by stakeholders was considered a prerequisite for successful implementation. Additional design principles included transparency, reproducibility of the surveillance results, achieving an optimized workload reduction, sustainability, privacy by design, and compliance with applicable laws and regulations. Further, the AS SSI systems should be based on available routine care data and aligned with (international) standards where possible.

A classification algorithm for semi-automated surveillance of deep SSI after hip and knee replacement was developed and validated in Dutch hospitals [15, 27, 28, 35]. All stakeholders agreed to exclude superficial SSI from surveillance.

Central coordination of AS SSI was considered essential to ensure the comparability of surveillance results between individual hospitals and comparability over time—a requirement for benchmarking. A local implementation approach with every hospital developing its own automated surveillance system and subsequently sharing surveillance outcomes with the RIVM was considered feasible and preferable due to the heterogeneity of source data, the possibility of semi-AS, workload reduction, and the lack of need to share large amounts of data. Furthermore, this approach enables participating hospitals to add their local requirements to the automated surveillance system used.

### Requirements, data sources and definitions

Availability of routine care data for reuse in surveillance activities was explored. Acceptance criteria for the algorithms have been defined to allow for deviations from the algorithm by individual hospitals due to differences in data availability between hospitals, under the conditions that they are justified and validated.

Furthermore, algorithm and data specifications have been defined to ensure comparability of surveillance results. This also includes the development of a minimum list of requirements for an AS system by AS and IT experts from the RIVM and hospitals. Using this approach, the design principles mentioned above such as quality and comparability of surveillance results, transparency, and sustainability of surveillance methods as well as compliance with laws and regulations could be ensured.

Data sources are provided by HIS including EHR and LIMS (Table 2).

### Implementation, maintenance and operationalization

In 2022, implementation of AS SSI was started in five frontrunner hospitals. At the same time, a parallel implementation evaluation study was conducted to learn from pitfalls that might arise and to improve the implementation strategy. After two years, three of these hospitals succeeded and several more hospitals will shortly follow.

To achieve sustainable automated surveillance, it is essential to facilitate the implementation and maintenance of local automated surveillance systems [8]. For this purpose, a manual, user group meetings, individual guidance, and training (currently developed as an e-learning collaboration with the University Medical Hospital Utrecht) were developed [36].

### Obstacles and challenges

Results from the evaluation study showed that implementation could be improved by supporting communication between hospitals to encourage the exchange of knowledge and experience of the process [37]. Furthermore, a considerable investment in time, especially by IPC and IT specialists, is required. It is a challenge to make this a high priority and to reach sufficient capacity in the departments involved, most importantly, the IT department. This highlights the importance for successful implementation of good local project management, commitment at higher management levels, and the appropriate assignment of roles and responsibilities.

Maintaining central oversight is not only a challenge for the hospital, it is also one for the coordinating centre. Although a minimum list of requirements has been defined, external validation is limited due to the high variety in HIS and formats as well as the structure of the AS systems.

## Internal quality control and improvement: one application of the AS SSI system from Bellvitge University Hospital (Spain)

### Purpose of surveillance and the surveillance population

The Bellvitge University Hospital (Spain) is a 700-bed teaching hospital located in the southern metropolitan area of Barcelona (Catalonia, Spain) and acts as referral centre for more than two million people who require highly complex care. The hospital developed a semi-AS SSI for internal quality control and improvement. Frontline stakeholders (IPC teams, surgeons, nurses) and the hospital management benefit from more accurate surveillance data as it enables them to monitor trends, identify areas for improvement, and guide quality improvement activities such as peri-operative antibiotic prophylaxis or post-operative wound care. This represents an example of a locally implemented AS developed by the hospital itself. Automation aims to reduce the workload for performance of surveillance. Adult patients undergoing cardiac surgery and prosthetic knee and hip replacements at a single hospital represent the surveillance population (Table 2).

### Design principles, implementation approach, selection of algorithm, and level of automation (full/semi)

Data specifications rely on the identification of the population under surveillance (denominator data) through the selection of specific ICD-10 codes. Currently, completeness and reliability of data extraction is being verified by its comparison with manual surveillance records. AS SSI has been locally developed and implemented, and the hospital requirement to report to the Catalan Surveillance program of healthcare associated infections in Catalonia (Vigilància de les Infeccions Relacionades amb l'Atenció Sanitària a Catalunya, VINCat) is being met. Within this surveillance program, standardized criteria are used to obtain data comparable between hospitals. Furthermore, surveillance needs as well as quality improvement objectives as defined by the management board are taken into consideration [38]. The IPC team and IT personnel in collaboration with microbiologists, pharmacists, and the healthcare coding department were involved in developing the design principles of extraction. For the identification of numerators (namely SSIs), the algorithms for semi-automated AS SSI as previously described by van Rooden et al. are applied [27] with some minor modifications of its components/definitions according to the feasibility of data extraction in this setting.

Principles that were followed to achieve accurate and reliable data extraction have been reported in the Supplementary material.

The implementation strategy includes maintenance of a comprehensive version control and documentation that summarizes design decisions, algorithms, and methodologies that are important to facilitating the understanding, reproducibility, and scalability of the methodology for other institutions. Furthermore, it is recommendable that the data management plan is well-prepared to explain how to collect, store, and share the data (if necessary).

### Requirements, data sources and definitions

The first step of AS development entailed the specification of standardized definitions that are clear and consistent. This was crucial for facilitating a common understanding of the methodology and interpretation of the final results (metrics) by different stakeholders of the organization. Definitions include algorithm components as well as inclusion and exclusion criteria of SSI surveillance. In a second step, an accurate measurement of patients under surveillance (as denominator) and patients with SSI detected within a defined timeframe (as numerator) was necessary. To achieve this, adequate data sources for AS design and development needed to be identified and selected. Routine-care data was extracted from the Minimum Basic Data Set (CMBD in the Spanish acronym) that contains administrative, socio-demographic, and clinical data from hospital discharge summaries from healthcare centres that are part of the Spanish National Health System. Furthermore, microbiological, radiological, and pharmacy data are obtained from the hospital data warehouse. Such data also assumes requests for the results of certain examinations, e.g. computer tomography (CT).

The algorithm is a simple decision-tree based on a combination of different hospital data including administrative information (e.g., hospital admissions), as well as microbiological, radiological, and pharmacy data. Using this information, patients are classified into patients with a high or low probability of SSI. Patients classified with high probability of SSI, undergo manual chart review to confirm the diagnosis (semi-automation).

The last step of AS SSI development on the hospital level required validation of the “automated process” by comparing the detection of patients at risk (denominator data) and patients that acquired SSI (nominator data) with data from conventional (manual) surveillance, or real-world data. A detailed analysis of discrepancies was performed to understand reasons for any AS misclassification.

### Implementation, maintenance and operationalization

At present, the implementation phase is ongoing. Thus, AS SSI still co-exists with manual surveillance. By the end of 2024, the manual surveillance will be abandoned after successful validation of the methodology with one year of prospective data.

One frequently reported obstacle to successful implementation of AS SSI is a lack of staff engagement due to difficulties accepting the new surveillance methodology [39]. To overcome this and increase stakeholder buy-in, the team at Bellvitge hospital involved surgeons and nurses in AS development from the beginning. Furthermore, hospital leadership was informed regularly on the benefits of AS surveillance for the institution. These benefits included decreased workload as well as better data quality and standardized output data that increases comparability over time. The former can also increase acceptance of AS SSI significantly.

SSI surveillance, regardless of manual or automated execution, is longitudinal and is intended to take place over extended periods of time. Thus, maintenance and regular audits of the process are required to ensure sustained data quality. This includes annual monitoring to check whether data extraction and algorithm performance are still accurate. This is necessary as ICD-10 releases periodic updates that might compromise adequate detection of patients under surveillance. In addition, data sources used for the algorithm might change and if so, data extraction should be modified accordingly. For example, the microbiology department at Bellvitge University Hospital changed its database and the codes used during AS SSI development. Consequently, the microbiology component of the automated process was no longer extracted and some SSI were not detected. Moreover, surveillance definitions and clinical practices may vary over time and automated processes need to be adapted to these changes.

### Obstacles and challenges

The main obstacles to successful implementation of AS SSI at Bellvitge hospital were related to IT aspects. A close collaboration with the IT department is the key for effective development and implementation of AS SSI. However, it might sometimes be difficult to find periodic/continuous IT support with enough dedicated time to develop and keep the AS SSI updated. Furthermore, the linkage of information from different data sources proved challenging. Information silos were found distributed throughout the hospital data warehouse that were particularly difficult to overcome. Another challenge was the fact that the accuracy of procedural/diagnostic codes was highly dependent on the information reported in the surgical and hospital discharge reports. In this setting, coding errors were minimal [40]. However, they need to be double-checked before implementation.

During the process of automation, the main challenge was that manual surveillance represented an “imperfect gold standard” [7]. Human errors in manual surveillance, such as transcription errors or mistakes on inclusion criteria, were discovered after it was compared with automated surveillance data. Furthermore, surveillance was interrupted during the COVID-19 pandemic leading to incomplete manual surveillance reports. This “imperfect gold standard” made the process of validation with conventional manual surveillance more challenging and confirmed the experience in other studies [16, 27, 40].

In summary, quality control is the key to obtaining accurate and reliable results for decision-making, thus mitigating the risks over time even in a setting with changing circumstances. A multidisciplinary team of frontline stakeholders (IPC team, surgeons, nurses), hospital leadership, and IT personnel is necessary to guarantee successful development, implementation, and maintenance of AS SSI.

## Research: one application of the French AS SSI system (SPICMI)

### Purpose of surveillance and the surveillance population

In 2018, the national agency Public Health France named the regional Centre for Prevention of HAI (CPias) to manage SPICMI (Surveillance and Prevention of Infection in Surgery and Interventional Medicine), a new national SSI surveillance program. CPias is a public institution which supports the Regional Health Agencies (ARS) responsible for the surveillance and prevention of HAI.

First, SPICMI facilitates a semi-automated and locally implemented process based on hospital routine databases. Thereby, it helps support healthcare facilities (HCF) using data from their own local IT systems. Second, the program can be used to evaluate trends in SSI incidence and the effectiveness of SSI prevention measures applied, in addition to surveillance, for example, of the effect of skin preparations or antibiotic prophylaxis [41, 42]. The SPICMI program also works to determine the optimal patient case mix to be used for hospital benchmark comparisons.

The purpose of automation in SPICMI was time saving in data collection and the improvement of data sensitivity [22, 43, 44]. The SPICMI program was launched following experience with an older AS SSI system called ISO-ORTHO that was in place from 2018 to 2020. ISO-ORTHO was fully automated and had been centrally implemented. It was focussed on total hip arthroplasty or total knee arthroplasty (THA/TKA) [45]. Here we will focus on the surveillance purpose “Research” illustrated by a modelling study conducted in 2021 [13]. The study analysed a subgroup of a surveillance population covered by SPICMI. It included patients undergoing at least one of 16 selected surgical procedures at 27 participating HCFs [13]. The aim of this modelling study was to assess the performance of a risk-adjustment model for reporting and benchmarking SSI. This model was based on 6 comorbidities, and utilized a set of variables extracted from the hospital discharge database (HDD). Subsequently, the performance of this model was compared with models that combined variable sets from various data sources [13].

### Design principles, implementation approach, selection of algorithm and level of automation (full/semi)

SPICMI represents a semi-automated, locally implemented AS SSI [13]. Its goal is to fulfill design principles established by PRAISE, such as high standardization, transparency, and sustainability. The program is based on voluntary participation of hospitals. It assumes that participating hospitals will select one or more surgical wards for yearly surveillance. They are also to select at least one surgical specialty, and at least one associated procedure of 16 targeted procedures for inclusion during the first 6 months of the year [46]. This approach assumes that each participating hospital will provide sufficient human resources and IT expertise for processing the data, which, unfortunately, is not always the case. Design principles, the implementation approach, and the selection of SPICMI algorithms also applies to the modelling study [13] that illustrates the surveillance purpose “Research” here.

### Requirements, data sources and definitions

SPICMI database implementation required assurances to the French Commission for Information Technology and Liberties (CNIL) that data protection regulations would be met. Using SPICMI data for research purposes requires anonymization of individual patient data. This important prerequisite was met by Picard and colleagues who evaluated the performance of different comorbidity-based risk-adjustment models for SSI reporting and benchmarking using variables from different data sources [13].

In SPICMI, the algorithm for SSI detection was based on data extracted from the local information system including three main data sources: electronic health records represented primarily by the hospital discharge database (HDD) for procedures and diagnosis (ICD-10 codification), microbiology lab data, and antibiotic prescriptions (urology only) [46]. If available, non-structured electronic clinical data records could be also used for SSI diagnosis confirmation. Details of SSI definition (ECDC/CDC based [47, 48]) can be found in the Supplementary material.

Two types of monitoring were proposed for SPICMI to provide a sustainable surveillance tool for HCF participation: The “lighter” unit-based monitoring included sharing data on patients with SSI (numerator) and aggregated data on all procedures performed in the facility for the target specialty(s) (denominator). The “heavier” patient-based monitoring included all data on SSI and risk factors including National Nosocomial Infection Surveillance System (NNIS) components and comorbidities extracted from HDDs.

Data extracted from HDD included age, sex, length of preoperative stay, any outpatient surgery and comorbidities, such as cancer [49, 50], diabetes [51–54], arterial hypertension [55], obesity [51, 53–56], malnutrition [56], and immunodeficiency [57]. In the modelling study presented here to illustrate the surveillance purpose “Research”[13], additional risk index factors were extracted from other databases, such as operating room databases [58]. They included the ASA (American Society of Anesthesiologists) anesthetic risk score, Altemeier wound contamination class, duration of the surgical procedure, and its classification as emergency or elective [13].

### Implementation, maintenance and operationalization

Prior to SPICMI implementation, a pilot survey was conducted to evaluate the status of readiness of the local IT systems in place. The study showed that the current status of local IT systems were very heterogeneous among hospitals and relied primarily on home-made software developed at the HCF or purchased from various software providers. At that time, only part of HCF had the capacity to implement automated surveillance.

As part of this semi-automated surveillance, the CPias created an electronic (e)-platform that enabled hospitals to record and import their data [59]. Additional details can be found in the Supplementary material. Once the program had been successfully implemented, each HCF could download automated reports and display individual files locally. Data and analyses at the e-platform level were stored on a server that was legally authorized to manage health data (with the approval of the National Commission for Information Technology and Liberties).

As part of the determination of the patient case mix for hospital benchmarking, a pilot study was conducted that tested whether risk-adjustment of SSI to comorbidities extracted from common HDD was comparable with NNIS or NHSN risk components. In conclusion, the electronic HDD-based comorbidities model was comparable to other models for patient case mix while being more commonly accessible than other data sources for most hospitals in France [13].

### Obstacles and challenges

In 2023, a survey on user satisfaction was sent to all SPICMI participants. The user feedback identified a need for more training and teaching materials. In response, targeted webinars, updated tutorials, and a section with answers to frequently asked questions (FAQ) were provided on the e-platform [59]. Obstacles were also experienced in data collection, especially when HCF did not have comprehensive software training or experienced a shortage of IT staff. Furthermore, another important issue was raised by incompatible software systems that required manual data compilation. Technical challenges, including the requirement to generate a formatted Excel® file from multiple data sources and upload these files to the e-platform, were further obstacles. Errors that occurred included the coding of some variables extracted from the HDDs that were not correctly filled in, which required post-hoc recoding and sometimes manual editing of patient data. Close collaboration with IT specialists is required to obtain an initial dataset from the HDD and must not be an obstacle for local teams.

## General practical steps from conception to implementation

**Fig. 1:**
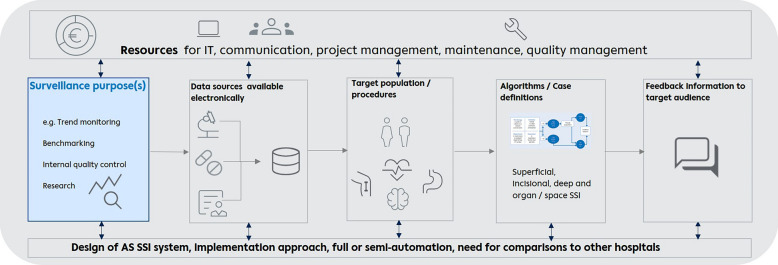
Overview of decisions to be made during development and implementation AS SSI systems. Visualization of algorithms adapted from [37]

An overview of decisions to be made for implementation of AS SSI systems is visualized in Fig. 1. Items relevant to reporting decision making in the development of an automated surveillance system are summarized in Supplemental Table 1. The first decision that is needed by stakeholders who are implementing AS SSI in their hospital or surveillance network is determining which surveillance purposes should be addressed by their AS SSI system. This is pivotal for the performance of AS SSI and its setup. However, the design of an AS system and specific choices will also have to be tailored to the data that is available electronically and suitable for use in AS SSI. In Denmark all patient data is available in uniform databases. Consequently, the AS SSI system HAIBA in Denmark was centrally implemented and highly standardized algorithms have been developed [29]. This AS SSI system is constantly managed by the National Public Health Institute. A central approach requires fewer resources in individual hospital IT departments. In addition, a certain level of commitment on the part of local IT teams is always essential to ensure maintenance and adherence to local requirements. As for the national public health institute, this central approach requires a reasonable amount of worktime and attention to guarantee data security, periodic updates, and interaction with the hospital’s IT and IPC departments. Less centralized approaches may be preferred because of concerns about data protection and governance. For example, the Dutch Public Health Institute (RIVM) designed an AS SSI system for hip and knee replacement (PAS ORTHO) using a local implementation strategy. In this system, hospitals are allowed to deviate from the standardized algorithm when following well-defined criteria. Each HCF uses an individually tailored algorithm developed by hospital IT teams that considers local situations based on the RIVM protocol with its data specifications and minimum requirements for the AS system. Subsequently, each hospital shares surveillance results with the Dutch Public Health Institute. RIVM generates aggregated and stratified data to facilitate benchmarking. The main reason for this local approach was the fact that it is less vulnerable in terms of data protection issues. However, under such a regime each hospital needs to provide dedicated IT personnel for development and maintenance of these algorithms. In France, the decision to use a local implementation approach and semi-automation for SPICMI was based on previous experience with ISO-ORTHO. This fully automated AS SSI system for total hip arthroplasty or total knee arthroplasty system was in place from 2018 to 2020 [45]. ISO-ORTHO was able to provide hospitals with a metric for SSI assessment [45], but IT resources on the hospital level were highly heterogenous and only a small part of HCF had the capacity to implement fully automated surveillance at the time. As a consequence, a semi-automated and locally-implemented AS SSI system (SPICMI) was chosen.

Using a central or local implementation approach is highly dependent on the decision of the level of automation which in turn is dependent on data sources, algorithms and definitions used. Fully automated AS SSI systems have been reported for HAIBA in Denmark that focus on hip or knee procedures [29, 45], while semi-automation is used for PAS-ORTHO in the Netherlands, SPICMI in France, and the AS SSI system at Bellvitge hospital in Spain. Full as opposed to semi-automation might be associated with reduced perception of data ownership by users since surgeons and IPC staff are not actively involved in case identification. At the same time, workload reduction was reported as a goal of automation for most AS SSI systems and this is higher with full automation. The pros and cons of fully and semi-automated surveillance were discussed earlier [7]. Semi-automated surveillance has been demonstrated to significantly reduce the workload of the IPC staff responsible for manual surveillance from almost 60% to 98%, depending on the type of surgery [16, 28, 60].

Regardless of a centrally or locally organized approach, the design of AS SSI should be as simple as possible to increase comparability. Ideally, a modular conception will be developed that requires a minimal dataset (MDS)—both for the algorithm and for data collection—that can be broadened with additional data. The MDS needs to include basic information such as demographic patient data and information on procedures as well as medical data, preferably defined by (international) standard terminology. More homogeneous AS SSI systems in Europe may one day even enable meaningful comparisons to be made at the international level.

Additional information can improve interpretation of AS SSI. Such information may include data for risk stratifications, such as comorbidities, or other relevant data, like peri-operative prophylaxis or post-operative wound care. Basic principles of data storage, data structure and standardization, as well as interoperability and algorithms must be followed [34].

The challenges and obstacles reported largely overlapped all AS SSI systems. All experts agreed that time, commitment, and the resources of IT specialists are essential and represent the most important obstacles. Obviously, the most important prerequisite for any form of automation is the availability of electronic data that can be automatically processed. This has been confirmed by a recent German survey on the status of digitalization for SSI surveillance systems that identified a large degree of heterogeneity among surgical departments in Germany [61]. The authors concluded that improving availability and accessibility of information in HIS and meeting interoperability standards are important prerequisites [61]. The intention to introduce automated surveillance has been around for many years. However, it is still hampered by limited data available in a suitable format, by very complex clinical environments requiring the involvement of many stakeholders, and by a large variety of procedures. To start with, HCF have very diverse and fragmentary IT environments for patient health records. The electronic infrastructure present in HCF includes administrational IT-systems, financial IT-systems, complex logistic software products, laboratory databases, and HIS, as well as EHR. In most cases, the data structure and architecture of these databases are highly variable, ranging from uniquely structured data to free text. To sum up, IT infrastructure often lacks interoperability of different databases. Furthermore, automation shifts workload from manual surveillance done by IPC staff to interdisciplinary teams that involve IT and IPC specialists, clinicians, and microbiologists. There has been a consensus among all experts regarding solutions suggested and applied to overcoming these obstacles. They have suggested adequate and permanent resources, good communication, as well as early involvement of end-users and adequate commitment by the management level, IT departments, IPC teams, and clinicians as key to successful AS SSI implementation. Consequently, IPC teams will require new expertise, including interdisciplinary communication and project management skills. The well-known fact that communication is a major pillar of healthcare and IPC becomes even more important [62]. For example, the Danish HAIBA system’s personal communication with end-users revealed that healthcare professionals found it difficult to relate data from aggregated levels to specific patient data [33]. Thus, communication tools should include training for clinical staff on how to use, analyse, and interpret AS SSI data. Moreover, central support with practical tools and an exchange of experience between hospitals were considered important for users of the AS SSI system during implementation of PAS-ORTHO in PREZIES in the Netherlands.

## Discussion

The increasing amount of data that is electronically available provides optimal prerequisites for the implementation of AS SSI in hospitals and surveillance networks. However, a clear view of the concrete steps to be taken and decisions to be made when starting development of AS SSI is often lacking, both on the hospital and the surveillance network level. The PRAISE SSI working group decided to fill this gap by providing practical recommendations for hospitals and surveillance networks that planned to implement AS SSI. This article focuses on four different surveillance purposes in AS SSI: trend monitoring, benchmarking, internal quality control/improvement, and research. For each surveillance purpose, basic requirements are described and illustrated by detailed descriptions of successful examples of AS SSI systems already in place that highlight the potential of this approach.

Numerous publications worldwide have shown AS SSI to be highly accurate, specific, sensitive, reliable, feasible for implementation, and of great potential for reducing workload [15–28]. In Europe, numerous activities are underway to develop, implement, and use AS SSI in hospitals and public health institutions. Successfully implemented AS SSI will enable HCF to easily monitor performance, do benchmarking, increase awareness of SSI, and eliminate deficits that may increase the risk of acquiring an SSI [63, 64].

Previous work by Verberk and colleagues was not focused on SSI but characterized three AS systems for HAI that were in use in Europe at the time (HAI-Proactive from Sweden, semi-automated SSI surveillance from Utrecht University Medical Centre (UMCU) in the Netherlands and HAIBA from Denmark) [11]. The authors identified heterogeneity among these systems from reports on hands-on experience with implementation, maintenance, clinical needs, and feedback [11]. Here, we address only one HAI, namely SSI, and found both—similarities and differences—between the AS SSI systems characterized. Most operators reported identical reasons for automation (workload reduction), focused on orthopaedic procedures for their AS SSI systems and named transparency and sustainability as design principles. The differences identified between the AS SSI systems highlight the fact that decisions made during implementation are not only dependent on the HAI type targeted (e.g. hospital-onset bacteraemia, urinary tract infection, or SSI), but also on the setting and goal of surveillance (surveillance purpose). AS SSI systems differ in terms of setting (single hospital, hospital network, or even nationwide), target population (patient groups and procedures), the implementation approach (centrally or locally implemented), level of automation (semi or fully automated), data source systems applied, and SSI types (all SSI or deep/organ SSI).

Successful implementation is not only dependent on the decisions related to case definition, source data, and algorithms. Operators of AS SSI systems agreed on the following actions by stakeholders and decision-makers required for successful implementation and maintenance of AS SSI systems in hospitals and surveillance networks:

1. Give AS SSI high priority by providing a clear mandate from higher management levels in the hospital/surveillance network/country or even a broader scale. This includes appropriate allocation of roles and responsibilities in all departments and among all players involved.
2. Provide permanent resources for IT support to assure successful implementation, but also maintenance and sustainability of AS SSI systems.
3. Provide permanent resources for project management and intersection teams for AS SSI. They are in charge of enabling close and constant communication and early involvement of clinicians (end-users), IPC teams, and IT experts; providing training for clinicians (end-users) on data application for IPC as well as organizing regular maintenance meetings.
4. Preferably, support databases that allow interoperability and are compatible between hospitals, regions, and, if possible, even countries throughout Europe.

## Strength and limitations

This study aimed to provide practical advice and to share experience with existing AS SSI systems. It focusses on four different surveillance purposes. Toward this end, information was provided by actual operators of existing AS SSI systems. Even though this approach has a high risk of bias and is not always grounded on scientific evaluation studies, this article contains valuable information for practitioners that is usually not included in research articles. As such, recommendations could not have been based on a study of the literature.

Decisions on which surveillance purposes, which AS SSI systems as practical examples, and which items for reporting them (in Verberk’s sense [11]) should be included in this article were based on consensus reached during expert discussions in PRAISE SSI working group meetings held between 2023 and 2024.

This work involved working group members from more than 15 institutions and 10 countries in Western Europe suggesting high representativeness and completeness for European high-income countries. At the same time, our work does not include information from high-income countries outside Europe or from any middle- and low-income countries. However, results may also apply to other countries given that electronic routine care data is available for reuse.

## Conclusions

In conclusion, implementation of AS SSI requires a number of decisions concerning the purposes of surveillance, the data sources, implementation strategies, methodology, and much more. Clarity on the intended application (e.g. surveillance purpose) is essential for guiding decisions on the implementation strategy. Limited commitment and a lack of resources for IT staff were identified as main obstacles for the development and use of AS SSI systems. Thus, stakeholders who plan to implement AS SSI in their hospital or surveillance networks should provide adequate resources for IT specialists, project management, and for a communication infrastructure that allows regular exchanges between the management levels, IT departments, IPC teams, and clinicians. This is crucial to ensuring commitment on the part of all players involved to the successful and sustainable implementation of AS SSI.

## Supplementary Information

Additional file1

## Data Availability

No datasets were generated or analysed during the current study.
