# Supplementary material for: Automated surveillance for surgical site infections (SSI) in hospitals and surveillance networks–expert perspectives for implementation
Source: Antimicrob Resist Infect Control. 2024 Dec 23;13:155. doi: 10.1186/s13756-024-01505-2 (PMC11667888; doi:10.1186/s13756-024-01505-2)
Supplement: Supplementary file 1 — Additional file1 [file 13756_2024_1505_MOESM1_ESM.docx]

**Supplementary material**

**Members of the PRAISE SSI working group involved in generation of Table 1, in alphabetical order:**

1. Mohamed Abbas, Infection Control Programme and WHO Collaborating Centre on Infection Prevention and Control and Antimicrobial Resistance, Geneva University Hospitals, Geneva, Switzerland and MRC Centre for Global Infectious Disease Analysis, Jameel Institute, School of Public Health, Imperial College London, United Kingdom
2. Hoger Amin, Department of Data Integration and Analysis, Staten Serum Institut, Copenhagen, Denmark
3. Seven Aghdassi, Institute of Hygiene and Environmental Medicine, Charité Universitätsmedizin Berlin, corporate member of Freie Universität Berlin, Humboldt-Universität zu Berlin and Berlin Institute of Health, Berlin, Germany, Berlin Institute of Health at Charité - Universitätsmedizin Berlin, BIH Biomedical Innovation Academy, BIH Charité Digital Clinician Scientist Program, Berlin, Germany
4. Pascal Astagneau, Centre for prevention of healthcare-associated infections, Assistance Publique, Hôpitaux de Paris, France and Institute of Epidemiology and Public Health, INSERM, Sorbonne University, Paris, France
5. Isabelle Arnaud, Centre for prevention of healthcare-associated infections, Assistance Publique - Hôpitaux de Paris, France
6. Manon Brekelmans, Department of Medical Microbiology and Infection Control, University Medical Center Utrecht, the Netherlands and Centre for Infectious Diseases Control, National Institute for Public Health and the Environment, Bilthoven, the Netherlands
7. Heike Düsseldorf, Department of Hospital Epidemiology and Infection Control, Medical University of Vienna, Vienna, Austria
8. Sophie Gubbels, Department of Data Integration and Analysis, Staten Serum Institut, Copenhagen, Denmark
9. Karl Mertens, Service of healthcare-associated infections and antimicrobial resistance, Sciensano, Brussels, Belgium
10. Maaike S.M. van Mourik, Department of Medical Microbiology and Infection Control, University Medical Center Utrecht, the Netherlands
11. Mireia Puig-Asensio, Department of Infectious Diseases, Bellvitge University Hospital, L’Hospitalet de Llobregat, Barcelona, Spain, Centro de Investigación Biomédica en Red de Enfermedades Infecciosas (CIBERINFEC¸ CB21/13/00009), Instituto de Salud Carlos III, Madrid, Spain
12. Stephanie van Rooden, Centre for Infectious Diseases Control, National Institute for Public Health and the Environment, Bilthoven, the Netherlands
13. Elisabeth Presterl, Department of Hospital Epidemiology and Infection Control, Medical University of Vienna, Vienna, Austria
14. Suzanne D. van der Werff, Department of Medicine Solna, Division of Infectious Diseases, Karolinska Institutet, Stockholm, Sweden and Department of Infectious Diseases, Karolinska University Hospital, Stockholm, Sweden

# Trend monitoring - application of the Danish Healthcare-associated infections database (HAIBA)

## **Implementation, maintenance and operationalization**

In the future, the Regional Clinical Quality Programme (RKKP) intends to implement a similar quality indicator for knee arthroplasty surgeries based on HAIBA’s SSI surveillance data.

# Benchmarking - application of the Dutch AS SSI system after orthopedic surgery (PREZIES PAS ORTHO)

## **Requirements, data sources and definitions**

Organization of data governance was a crucial step in this process. An automated surveillance module could be integrated in the PREZIES network and its governance structure; in addition, roles and responsibilities of the RIVM and hospitals were described, given the local implementation strategy (in line with the PRAISE Roadmap, refer to table 6) (1).

# Internal quality control and improvement - application of the AS SSI from Bellvitge University Hospital (Spain)

## **Design principles, implementation strategy, and selection of algorithm (fully / semi**)

The following principles are applied to achieve an accurate and reliable data extraction: i) Structured data are used to increase robustness of the process, avoiding the use of too specific criteria that could make the methodology less adaptable to clinical or coding changes over time. Such structured data include e.g. ATC code J01 for antibiotics or cultures sent to the microbiology department. ii) A minimal data set (MDS) and standardized surveillance definitions were applied to obtain reproducible results overtime. As recently reported, deviations in algorithms and clinical definitions are possible at the hospital level dependent on the local clinical practices and feasibility of data extraction [1]. When initial algorithm definitions are modified, re-validation of the new data extraction and algorithm performance becomes necessary. Iii) An extraction method that accounted for multiple procedures during surgery and combines different procedure codes was developed. This is essential to ensure data accuracy, e.g. if SSI surveillance is not intended to imply combined surgeries such as cardiac aortic valve and aortic root replacement. In this scenario, different procedure ICD-10 codes were considered. Data extraction included the surgical procedure code '02RF0JZ'(aortic cardiac valve replacement), but not the code '02RX0JZ' (for ascending thoracic aorta).

# Research - application of the French automated SSI surveillance system (SPICMI)

## **Requirements, data sources and definitions**

Data sources used are electronic health records mainly represented by hospital discharge database (HDD) for procedures and diagnosis (ICD-10 codification), microbiology lab data, and antibiotic prescription (urology only) (2). If available, non-structured electronic clinical data records could be also used for SSI diagnosis. Based on these data sources, SSI detection was classified in 3 categories according to a combination of 2 criteria: 1) reoperation during the index stay or rehospitalization and 2) positive microbiological sample from one of the three levels of the surgical site (superficial, deep or organ space). These two events should have occurred within the 30 days following the initial surgery (90 days in case of remaining implants) according to standard usual definition (ECDC/CDC based) *(3, 4)*). The SSI was defined as highly probable if 2 criteria were present and as mildly probable if only 1 criterion was present. In this case, the IPC team should confirm whether SSI diagnosis has met the standard definition criteria by searching data from the clinical records, and confirming results with the surgeon. SSI diagnosis was rejected if none of the two criteria were present. The same process was used for urological surgery based on either positive urine culture and/or antibiotic prescription more than 48h within the 30 days after surgery.

Three classes of SSI were defined including superficial SSI of the incision, deep SSI or organ / space SSI (3, 4).

a) Superficial SSI of the incision: infection that affects the skin and subcutaneous tissues around the incision during the first 30 days after surgery, AND at least one of the following criteria:

1. Purulent discharge at the superficial level of the incision with or without microbiological confirmation;

2. A microorganism isolated from a tissue or fluid culture, collected aseptically from the surface of the incision;

3. The patient has at least one of the following signs or symptoms: pain or tenderness, localized oedema, erythema, or local warmth of the wound associated with deliberate opening by the surgeon (or other clinician), unless if the culture is negative;

4. A superficial SSI of the incision diagnosed by the surgeon (or the clinician in charge of the patient).

b) Deep SSI: infection which appears to be linked to the procedure and which affects the deep soft tissues (e.g. fascia and/or muscle) of the incision during the 30 days following the procedure (or 90 days depending on the surgery), AND at least one of the following criteria:

1. Purulent discharge from the deep incision;

2. Spontaneous dehiscence of the deep incision, OR an incision deliberately opened or separated by the surgeon (or other clinician) when the patient has at least one sign or symptom of infection (fever [>38.5°C], localized pain or tenderness) AND when a micro- organism was isolated and collected aseptically from the soft tissues deep in the incision;

3. a deep abscess is identified by direct examination during re-operation, by histopathology or by radiological examination.

c) Organ/space SSI: infection that appears to be related to the procedure AND involves the surgical site organ/space (any anatomical part, other than the incision, opened or manipulated during the surgical procedure) that develops within 30 days of the procedure without implant (or 90 days depending on the surgery), AND at least one of the following criteria:

1. A purulent discharge from a drain placed in the organ/space;

2. An identification of microorganism(s) in a fluid or tissue sample collected aseptically from the organ/operative site space by a microbiological test based on culture or a method other than culture and having been carried out for diagnostic or treatment purposes;

3. An abscess in the organ/space is identified by a macroscopic approach during re-operation, by histo-pathological or radiological examination.

## **Implementation, maintenance and operationalization**

For the purpose of performing AS SSI, HCF must use their own data to complete a formatted Excel® file. Data export is done from the local HDD or other local IT databases that allows filling in all variables. For some variables that are extracted from other databases than the HDD databases, re-coding might be necessary

## **Barriers and challenges**

Since 2023, surgeons have been more and more involved in identification of codes from the HDD that are relevant for AS SSI. This approach particularly supports HCF with small local IPC teams to overcome hurdles that keep them from participating in the national network.

Since launching the SPICMI program, participation has gradually increased. Until today, 41.3% (n = 411) of French HCF practicing surgery participated at least once.

# General practical steps from conception to implementation

Supplemental Table 1: Items relevant to report for decision making in the development of an automated surveillance system

| **Items relevant to report for decision making in the development of an automated surveillance system** | |
| --- | --- |
| 1 | Surveillance purpose^1^ |
| 2 | Target audience^1^ |
|  | Aims of automation, design principles^2-3^ |
|  | Implementation approach^2^ |
| 3 | Data sources (selection surveillance population, algorithm, risk factors)^2-3^; MDS |
| 4 | Data qualifications and standardization^1-2^ |
| 5 | Surveillance population and inclusion criteria^1,3^ |
| 6 | Type of HAI and case definition^1-3^ |
| 7 | Semi/fully automated surveillance^1, 4^ |
| 8 | Algorithm (incl. performance measures) ^1-4^ |
| 9 | Validation (reference data, sample size) ^2, 3^ |
| 10 | Feedback information^1^ |
| 11 | Phase of implementation^3^ |
| 12 | Workflow, maintenance^3^ |
| 13 | Barriers/facilitators^3^ |

MDS, Minimal data set. Items as described by 1) Table 1, 2) van Mourik et al. 2021 (1), 3) Verberk et al. 2022 (5) and 4) van Mourik et al. 2018 (6).

# References

1. van Mourik MSM, van Rooden SM, Abbas M, Aspevall O, Astagneau P, Bonten MJM, et al. PRAISE: providing a roadmap for automated infection surveillance in Europe. Clin Microbiol Infect. 2021;27 Suppl 1:S3-S19.

2. SPicmi. Protocole de surveillance SPICMI 2024. Réseau de Prévention des Infections associées aux Soins. 2024 2024.

3. European Centre for Disease Prevention and Control (ECDC). Surveillance of surgical-site infections and prevention indicators in European hospitals: HAI net SSI protocol. 2017 2017. Report No.: version 2.2.

4. Centers for Disease Control and Prevention (CDC). National Healthcare Safety Network. 2022 NHSN patient safety component manual. 2022 2022.

5. Verberk JDM, Aghdassi SJS, Abbas M, Naucler P, Gubbels S, Maldonado N, et al. Automated surveillance systems for healthcare-associated infections: results from a European survey and experiences from real-life utilization. J Hosp Infect. 2022;122:35-43.

6. van Mourik MSM, Perencevich EN, Gastmeier P, Bonten MJM. Designing Surveillance of Healthcare-Associated Infections in the Era of Automation and Reporting Mandates. Clin Infect Dis. 2018;66(6):970-6.
